# Clinical spectrum of severe leptospirosis in the UK

**DOI:** 10.1099/jmmcr.0.000003

**Published:** 2014-12-01

**Authors:** Venkat Sivaprakasam, Wendy J Zochowski, Martin F Palmer

**Affiliations:** ^1^​ Department of Microbiology, Wye Valley NHS Trust, Hereford HR12ER, UK; ^2^​ Leptospira Reference Unit, Wye Valley NHS Trust, Hereford HR12ER, UK

**Keywords:** **Keywords:** biphasic illness, ceftriaxone, hepatorenal dysfunction, Weil’s disease, zoonosis.

## Abstract

**Introduction:**

Human leptospirosis is a global zoonotic infection with a characteristic biphasic illness and protean clinical manifestations. The majority are mild flu-like infections. The severe forms cause multiorgan damage with a greater predilection to hepatorenal failure.

**Case presentation:**

We attempted to analyse the clinical presentation of severe leptospirosis and decipher the clinical spectrum within this group by reviewing a series of 15 patients with leptospirosis requiring intensive care support for their management. We noticed complications becoming apparent before antibodies became detectable in the blood in a significant number of patients. This appears to belie the biphasic nature of leptospirosis and raises the question of whether the complications occur during the leptospiraemic phase or the immune phase of the infection. The presence of leptospiral DNA in the blood at this time as detected by a molecular assay strengthened this suspicion. Among the 15 patients with severe leptospirosis, only 3 (20 %) had an overseas travel history and the remaining 12 patients acquired their infection within the UK. Fourteen of the 15 patients had hepatorenal dysfunction, with seven requiring dialysis. Eight of the 15 patients received intravenous ceftriaxone with very good outcomes. Three showed significant clinical improvement after the administration of steroids.

**Conclusion:**

Many patients with severe leptospirosis will have complications on presentation. Molecular testing is now available for early diagnosis, facilitating early interventions. Ceftriaxone has been effective in treating severe leptospirosis. This study reminds clinicians to consider leptospirosis in the differential diagnosis of similar clinical spectra and offers tools for appropriate management.

## Introduction

Leptospirosis is a zoonosis of protean manifestations with a worldwide distribution (Levett, 2001). It is a biphasic illness with an initial febrile phase and a brief asymptomatic period followed by a secondary phase of illness (Turner, 1967). Most infections are subclinical or present with mild flu-like illness; this is known as the anicteric form (Levett, 2001). The icteric form is more severe with haemorrhagic episodes and multiorgan failure, and is known as Weil’s disease (Farr, 1995). There have been a few reports of a fulminant monophasic illness with rapid progression to hepatorenal failure, which may present in some as severe pulmonary haemorrhagic syndrome (Gouveia *et al*., 2008). Atypical presentations add to the challenge of diagnosis (Bal, 2005). Laboratory confirmation is by seroconversion or a fourfold rise in antibody titre on paired serum samples, although a single microscopic agglutination titre of ≥1 : 400 (Zochowski *et al*., 2001) would be indicative of infection with leptospirosis in a patient with compatible clinical signs and symptoms. Molecular methods detect leptospiral DNA in serum only in the early stages of the illness, and it disappears once antibodies become detectable at 5–7 days after the onset of illness. The UK National Leptospira Reference Unit in Hereford receives clinical samples from patients suspected of having leptospirosis from across England, Wales, Scotland and Northern Ireland. The definition of severe leptospirosis is undergoing significant change with a better understanding of its manifestations but still lacks clarity. For the purpose of this clinical review, we regarded a patient with a spectrum of illness compatible with leptospirosis along with laboratory confirmation and requiring intensive care management as having severe leptospirosis.

### Case report

We reviewed the clinical presentation of 15 patients with severe leptospirosis during a period of 21 months between 2011 and 2013. The clinical information provided in the epidemiology forms accompanying these samples was collated with additional information obtained by liaising with the respective clinicians. The limitations of serology in the acute stage of illness were resolved by a repeatable positive result from a multiplex real-time PCR assay (developmental assay), which was used as the major criterion for initial laboratory diagnosis in a few cases. All patients in this series except one had two or more serum samples collected for antibody testing, and the majority had a molecular assay performed on the acute sample. The results of these are shown in Table 1. Fourteen (93 %) were male; the age range was 21–68 years with a median age of 50 years. Three patients had a foreign travel history (two to France and one to South Africa) within the 4 weeks prior to onset of illness; the remaining 12 were resident in the UK at the time of their illness and all were involved in an activity with high risk of acquiring leptospirosis (Table 1). All required supportive care in an intensive therapy unit during their illness; some required ventilation, some needed inotropic support and many required dialysis. Most had one or more symptoms that characterize severe leptospirosis, either on onset or within 4–5 days of illness. It is possible that some may have had a preceding, unnoticed, subclinical or mild flu-like illness and thus fit the classical description of a biphasic presentation. Fourteen of the 15 patients had hepatorenal dysfunction, with seven requiring dialysis. One patient, whose renal function was normal, had a predominant central nervous system involvement with headache, photophobia and poor peripheral vision requiring ventilation for acute respiratory distress syndrome. The initial symptom was flu-like illness in 13 patients, with one having diarrhoea. The remaining two presented with myalgia and jaundice, respectively. The patient who presented with jaundice had a high serum bilirubin at 600 µmol l^−1^ and thrombocytopenia with platelets of 33×10^9^ l^−1^. Within 3 days of treatment with ceftriaxone, the bilirubin dropped to 184 µmol l^−1^ and platelets rose to 320×10^9^ l^−1^, followed by clinical recovery. There was no patient with any severe pulmonary complications specific to leptospirosis such as pulmonary haemorrhage in this case series, although three had acute respiratory distress syndrome. However, this does not exclude the possibility of alveolar haemorrhage, as there were no imaging results available. Complications relating to the central nervous system were seen in three patients ranging from hallucinations and photophobia to frank hepatic encephalopathy. The time from onset of illness to development of complications was less than 1 week in all patients except one, as shown in Table 1. In 11 of the 15 patients (80 %), it was within 5 days, and within this group, seven had a negative IgM ELISA result and a positive leptospiral DNA PCR (developmental assay) result. The serum sample taken during the acute stage of the illness was negative for leptospiral IgM antibodies and positive for leptospiral DNA by PCR in nine of the 15 (60 %) patients, thus allowing appropriate antimicrobial therapy to be initiated with good outcomes. This further supports our argument that the virulent effect of leptospiraemia as such contributes to the severity of the illness, leading to complications appearing in the earlier leptospiraemic phase rather than the later immune phase of the infection. Interestingly, none of the patients in this review showed a biphasic type of illness. This also highlights the importance of leptospiral PCR in the early diagnosis of acute infection and exposes the limitations of serology alone in the management of severe leptospirosis.

### Steroids as combination therapy

Patient 11 was a 48-year-old fisherman, admitted with a 5-day history of flu-like illness. He soon required dialysis with rapidly declining liver function and hepatic encephalopathy. He was transferred to a liver unit in preparation for liver transplant with a working diagnosis of fulminant immune hepatitis. *Leptospira* had been excluded with a negative IgM serology on the acute serum sample and, as there was no clinical information in the accompanying form, a leptospiral PCR was not done at that time. However, following a discussion with the clinical team, a leptospiral PCR carried out on the same sample was positive, which helped to remove him from the transplant list. The diagnosis of *Leptospira* infection was later confirmed by demonstrating seroconversion on a later sample. This patient was started on intravenous ceftriaxone along with steroids as an adjunctive therapy. He improved within 48 h with his serum bilirubin falling from 485 to 169 µmol l^−1^ and platelets rising from 86×10^9^ to 279×10^9^ l^−1^, and he was transferred to the medical ward within 48 hrs of the combined therapy starting. Interestingly patient 8, who was a 68-year-old man, faced a similar situation. He was admitted with jaundice and severe hepatorenal dysfunction and was short listed for a liver transplant. However, he gradually recovered without requiring a liver transplant.

Patient 9 was a 50-year-old male who worked in a pub and came into contact with sewage while clearing rain water. Two weeks later, he developed flu-like illness with myalgia and conjunctivitis and was septic on admission with a C-reactive protein level of 311 mg l^−1^ and white blood cell count of 15×10^9^ l^−1^. He was thrombocytopenic with a platelet count of 83×10^9^ l^−1^, and showed signs of acute renal failure with a high creatinine level of 636 µmol l^−1^ and high urea of 36.5 mmol l^−1^. He was treated with intravenous ceftriaxone and continued to have confusion, photophobia and hallucinations. It is not clear whether the increase in severity of illness after antibiotics was due to a Jarisch–Herxheimer-type reaction. However, he was started on prednisolone and responded significantly within a few days with the C-reactive protein level falling to 31 mg l^−1^ and platelets recovering from 83×10^9^ to 161×10^9^ l^−1^. Patient 10 was a 46-year-old male, who was treated with intravenous ceftriaxone and continued to have signs of increasing liver dysfunction. He was also treated with steroids with rapid clinical improvement.

Atypical symptoms were seen in a couple of our patients in this review. Patient 5, a 22-year-old male, was admitted to the acute medical unit under the gastroenterologist for fever and bleeding per rectum. He had been canoeing 2 weeks earlier and had an accidental fall into the river. Later, he became jaundiced and oliguric. He underwent dialysis and was treated with ceftriaxone for 10 days and subsequently made a full recovery. Patient 14, a 67-year-old male who kept birds and reported seeing rat droppings around his house, was treated in the intensive care unit for hepatorenal dysfunction with bilirubin at 677 µmol l^−1^, creatinine at 516 µmol l^−1^ and platelets at 50×10^9^ l^−1^. He developed epistaxis, and the bleeding from the nose stopped only after cauterization. He was discharged home after a week of ceftriaxone when his bilirubin decreased to 127 µmol l^−1^ and his creatinine to 197 µmol l^−1^, and his platelets rose to 324×10^9^ l^−1^.

## Discussion

Severe leptospirosis has been evaluated in previous studies by clinical and laboratory parameters, by association with pulmonary haemorrhage or based on clinical outcomes (mortality rate) (Dupont *et al.*, 1997). The clinical parameters range from hypotension, leucocytosis (>12 000 cells µl^−1^), chest symptoms, thrombocytopenia, oliguria, hyperkalaemia and cardiac arrhythmias (Ko *et al.*, 1999). In a recent review conducted in Greece, sequential organ failure assessment or SOFA scores were used to evaluate the outcome of severe leptospirosis requiring intensive care therapy (Velissaris *et al.*, 2012). In their review of 10 cases, the mortality rate was 30 % and they concluded that SOFA scores could assess severity but could not predict mortality in severe leptospirosis. In our review, all our patients had one or more, and in some all, of the clinical parameters used by others to characterize severity. Interestingly, all our patients had a favourable outcome except for one patient who had pre-existing co-morbidity prior to developing leptospirosis. The mortality rate in severe leptospirosis is reported to be 7–14 % in the hepatorenal failure group and could be as high as 40 % in those with haemorrhagic pneumonitis (McBride *et al.*, 2005). The mortality rate in this case series was 6.6 %, and severe pulmonary involvement was not seen. This is in stark contrast to an Indian study reviewing 60 patients requiring treatment on the intensive care unit, in which the mortality rate was as high as 52 % (Pappachan *et al.*, 2004). Another study in Brazil reported a similar mortality rate of 51 % in their cohort of 35 patients (Vieira & Brauner, 2002). A retrospective study conducted in New Caledonia reported 14 % mortality in their cohort of 71 patients with severe leptospirosis (Tubiana *et al*., 2013), and the definition of severity of illness in their study was different from that of ours. Nonetheless, the mortality rate in our review was only 6.6 %. The use of molecular techniques in diagnosing the infection at its early stages, thus facilitating early appropriate interventions, could explain our good clinical outcomes. The putative serogroup was identified as a probable Icterohaemorrhagiae by the MAT test in 4 patients (patients 4, 7, 11 and 13), and this serogroup is known to be associated with severe infections. However, as the serogroup could not be ascertained in the rest of the patients due to a lack of follow-up serum samples, it is difficult to draw any definite correlation between the severity of the illness and the infecting serogroup based on the findings of this review.

This review clearly demonstrates the usefulness of molecular tests in the early diagnosis of leptospirosis, which facilitate early management and avoid inappropriate therapies. The antibiotics used to treat severe cases of leptospirosis have ranged from benzyl penicillin and third-generation cephalosporins to piperacillin-tazobactam and meropenem. All patients in this report received different β-lactam antibiotics. Two studies conducted in Thailand demonstrated that there was no statistically significant difference in the clinical benefit between penicillin, ceftriaxone and cefotaxime in treating patients with severe leptospirosis (Panaphut *et al.*, 2003; Suputtamongkol *et al.*, 2004). However, eight of the 15 patients in our case report received intravenous ceftriaxone with very good outcomes. This, coupled with its once-daily dosing regime, makes it an ideal agent for treating patients with severe leptospirosis.

Several reports have indicated the benefit of the use of steroids in severe leptospirosis. One case report demonstrated the effect of a single bolus dose of methyl prednisolone combined with intravenous immunoglobulin, which produced rapid clinical improvement in a patient with multiorgan failure (Meaudre *et al*., 2008). Another case–control study in patients with severe leptospirosis who had pulmonary complications demonstrated a reduced mortality rate with methyl prednisolone therapy given initially as an intravenous bolus dose for 3 days and continued orally for a further 7 days (Shenoy *et al*., 2006). A further report on 30 patients with severe leptospirosis and pulmonary complications observed better outcomes with bolus doses of methyl prednisolone given along with antibiotics (Trivedi *et al*., 2001). A Sri Lankan study showed a significant reduction in mortality rates with methyl prednisolone in severe cases of leptospirosis (Kularatne *et al*., 2011). However, a recent prospective randomized controlled trial, involving 68 patients with severe leptospirosis with pulmonary complications, showed no significant benefit from using steroids (Niwattayakul *et al.*, 2010). In this review, three of our patients showed significant clinical improvement after the administration of steroids, as described previously. Although the literature has conflicting information on the benefit of steroid use in managing severe leptospirosis, our observations coupled with those of others merit more research in this area.

## Conclusion

- Many patients with severe leptospirosis will have complications on presentation.
- The diagnosis of human leptospirosis is now supported by molecular assays (PCR).
- Samples should be submitted at the earliest opportunity along with relevant clinical information including risk factors and the date of onset of symptoms.
- Samples taken within the first 5 days of illness, the window of seronegativity, are more likely to be positive by PCR, allowing prompt diagnosis and management.
- Ceftriaxone has been shown to be effective in treating severe leptospirosis.

**Table 1.** Serology results on acute and convalescent (conv) samples and PCR results along with risk activities and the time to complications of the 15 patients with severe leptospirosis

Patient no.	ELISA IgM titre		Microscopic agglutination test		PCR result	High-risk activities	Time to complications (days)
	Acute	Conv	Acute	Conv			
1	1 : 640	1 : 160	1 : 320	1 : 80	NT	Water skiing	3
2	−	1 : 2560	−	1 : 1280	DET	Kayaking	4
3	−	1 : 640	−	1 : 160	DET	Farmer	7
4	1 : 80	1 : 1280	−	1 : 5120	DET	Fall in ditch	11
5	1 : 160	1 : 2560	−	1 : 160	DET	Canoeing	1
6	−	1 : 2560	−	1 : 5120	DET	Watersports – South Africa	6
7	−	1 : 2560	−	1 : 640	NDET	Fishing – France	3
8	−	1 : 1280	−	1 : 320	DET	Occupational contact –waterways	6
9	1 : 160	1 : 2560	−	1 : 2560	DET	Flood water/sewage exposure	4
10	−	1 : 160	−	1 : 320	DET	Fishing – France	4
11	−	1 : 2560	−	1 : 640	DET	Fish farmer	4
12	−	1 : 640	−	−	DET	Traveller/rat exposure	3
13	1 : 640	1 : 1280	−	1 : 160	nt	Keeping chickens (probable rat exposure)	4
14	−	1 : 320	−	1 : 320	DET	Rat exposure	5
15	−	1 : 2560	−	1 : 160	DET	Water exposure	5

−, Negative; DET, leptospiral DNA detected; NDET, leptospiral DNA not detected; nt, not tested.
